# Direct effects of mast cell proteases, tryptase and chymase, on bronchial epithelial integrity proteins and anti-viral responses

**DOI:** 10.1186/s12865-021-00424-w

**Published:** 2021-06-02

**Authors:** Sangeetha Ramu, Hamid Akbarshahi, Sofia Mogren, Frida Berlin, Samuel Cerps, Mandy Menzel, Morten Hvidtfeldt, Celeste Porsbjerg, Lena Uller, Cecilia K. Andersson

**Affiliations:** 1 Department of Experimental Medical Science, Lund University, Lund, Sweden; 2 Department of Respiratory Medicine and Allergology, Lund University, Lund, Sweden; 3 Department of Respiratory Medicine, Bispebjerg and Frederiksberg Hospital, Copenhagen, Denmark

**Keywords:** Asthma, Mast cells, Tryptase, Chymase, Human bronchial epithelial cells

## Abstract

**Background:**

Mast cells (MCs) are known to contribute to both acute and chronic inflammation. Bronchial epithelial cells are the first line of defence against pathogens and a deficient anti-viral response has been suggested to play a role in the pathogenesis of asthma exacerbations. However, effects of MC mediators on bronchial epithelial immune response have been less studied. The aim of this study is to investigate the direct effects of stimulation with MC proteases, tryptase and chymase, on inflammatory and anti-viral responses in human bronchial epithelial cells (HBECs).

**Method:**

Cultured BEAS-2b cells and primary HBECs from 3 asthmatic patients were stimulated with tryptase or chymase (0.1 to 0.5 μg/ml) for 1, 3, 6 and 24 h. To study the effects of MC mediators on the anti-viral response, cells were stimulated with 10 μg/ml of viral mimic Poly (I:C) for 3 and 24 h following pre-treatment with 0.5 μg/ml tryptase or chymase for 3 h. Samples were analysed for changes in pro-inflammatory and anti-viral mediators and receptors using RT-qPCR, western blot and Luminex.

**Results:**

Tryptase and chymase induced release of the alarmin ATP and pro-inflammatory mediators IL-8, IL-6, IL-22 and MCP-1 from HBECs. Moreover, tryptase and chymase decreased the expression of E-cadherin and zonula occludens-1 expression from HBECs. Pre-treatment of HBECs with tryptase and chymase further increased Poly (I:C) induced IL-8 release at 3 h. Furthermore, tryptase significantly reduced type-I and III interferons (IFNs) and pattern recognition receptor (PRR) expression in HBECs. Tryptase impaired Poly (I:C) induced IFN and PRR expression which was restored by treatment of a serine protease inhibitor. Similar effects of tryptase on inflammation and anti-viral responses were also confirmed in primary HBECs from asthmatic patients.

**Conclusion:**

MC localization within the epithelium and the release of their proteases may play a critical role in asthma pathology by provoking pro-inflammatory and alarmin responses and downregulating IFNs. Furthermore, MC proteases induce downregulation of epithelial junction proteins which may lead to barrier dysfunction. In summary, our data suggests that mast cells may contribute towards impaired anti-viral epithelial responses during asthma exacerbations mediated by the protease activity of tryptase.

**Supplementary Information:**

The online version contains supplementary material available at 10.1186/s12865-021-00424-w.

## Background

Mast cells (MCs) are ideally positioned in the bronchial mucosa and epithelium to react to environmental factors, such as allergens and pathogens and thus initiate immunological responses. Their position within or near the airway epithelium leaves mast cells ideally situated to respond to inhaled allergens and pathogens, where they can promote an inflammatory airway response [1]. Numerous studies have demonstrated an increase in numbers of MCs in the asthmatic airway submucosa [2–7]. In addition, bronchial biopsies from patients with asthma show a significant increase in MC numbers within the bronchial epithelial layer [8, 9]. When MCs become activated, they degranulate and release mediators such as histamine, cytokines, proteoglycans and specific MCs proteases such as tryptase and chymase [10, 11]. However, they can also release smaller amounts of mediators through a process called piecemeal degranulation [12]. Tryptase and chymase are the most abundant serine proteases in the MC granules. Previous research has shown that mast cell proteases have both beneficial and detrimental roles in respiratory diseases [13–18]. Studies have reported increased MC infiltration and degranulation in the airways and lungs of allergen sensitized and challenged patients [19, 20] and higher levels of serum tryptase have been found in severe asthmatics compared to healthy individuals [21]. The role of MC proteases in relation to direct effects on bronchial epithelial cell production of pro-inflammatory cytokine release and anti-viral response is to a large extent unknown.

The bronchial epithelial barrier is an important feature of the innate immune system, providing protection against various environmental factors such as respiratory viruses. The immunological response to viruses begins with the recognition of a pathogen by pattern recognition receptors (PRRs) such as toll-like receptors (TLRs). These interactions lead to the production of numerous epithelial derived pro-inflammatory and anti-viral mediators [22]. Respiratory viral infections are a major cause of asthma exacerbations [23]. Bronchial epithelial cells from asthmatic patients have profound deficiencies in the production of anti-viral interferons in response to viral infection [24]. Treatment with anti-IgE and MC immunoregulatory therapy Omalizumab reduced virally induced asthma exacerbations and restored IFN levels indicating a role for MCs in asthma exacerbations [25–29]. Also, an altered barrier function can cause epithelial cell damage which can lead to increased epithelial permeability [30]. Asthmatics have been shown to have a disrupted epithelial barrier with a defective anti-viral response, although the mechanism behind this is unclear [29, 31–33].

In this current study, we investigated the effect of tryptase and chymase on inflammatory and anti-viral responses in HBECs. Our data showed that tryptase and chymase induce release of the metabolic alarmin ATP and that tryptase induced the pro-inflammatory mediators IL-8, IL-6, MCP-1 and IL-22 in HBECs. Furthermore, pre-treatment with tryptase and chymase enhanced Poly (I:C) induced IL-8 release, indicating an augmented inflammatory response in the presence of MC proteases. Additionally, MC proteases downregulated tight junctional molecules E-cadherin (E- CDH) and zonula occludens (ZO-1) associated with cell junction complexes in HBECs. Interestingly, tryptase significantly reduced viral induced type-I and III interferons (IFNs) and pattern recognition receptor (PRR) expression and this reduction was restored with a serine protease inhibitor. We further confirmed that tryptase reduced IFN- β in primary human bronchial epithelial cells from asthmatic donors.

## Results

### Mast cell proteases induce release of the alarmin ATP in bronchial epithelial cells

**Fig. 1:**
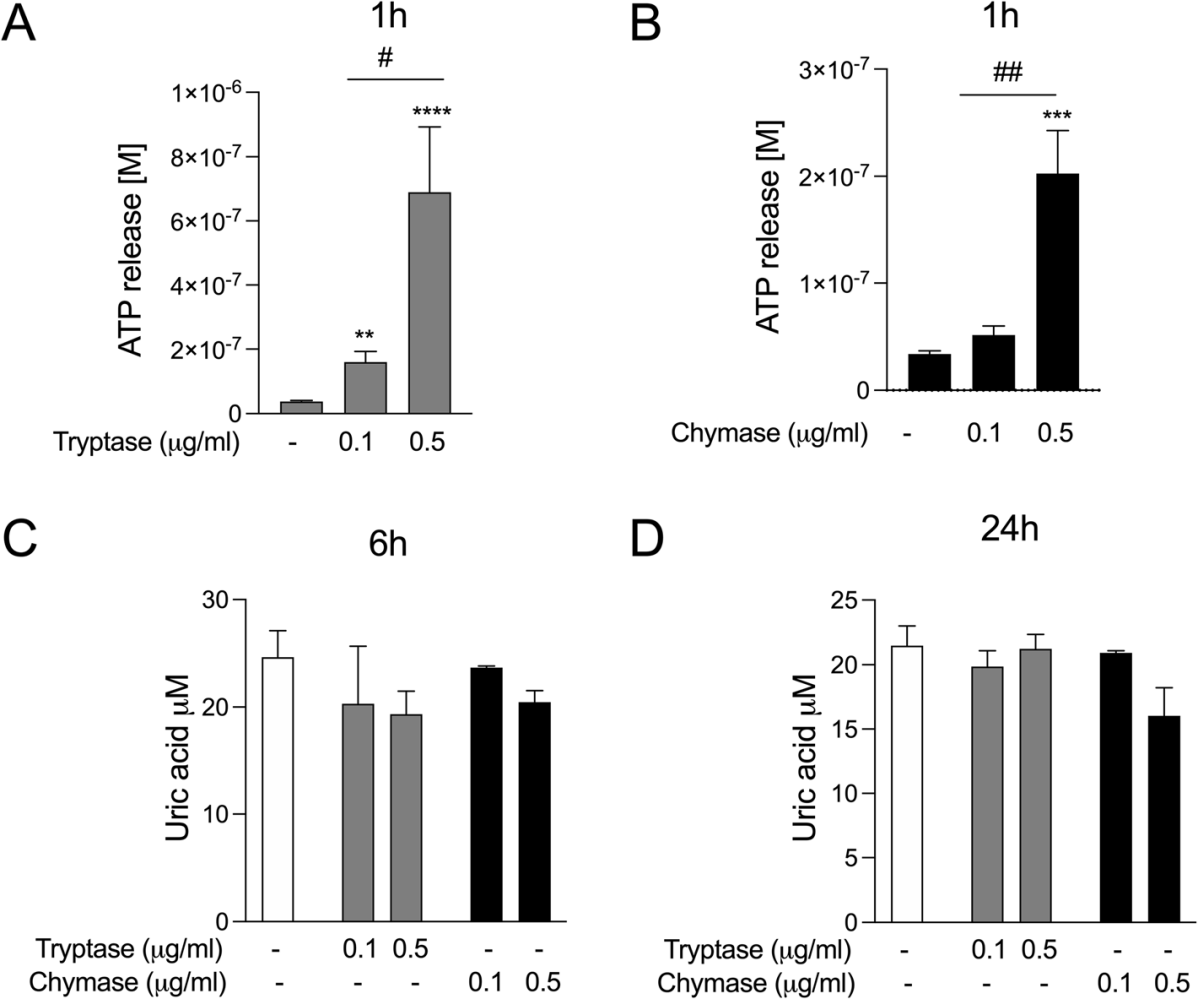
Mast cell proteases induce release of the alarmin ATP in HBECs. HBECs were treated with 0.1 μg/ml and 0.5 μg/ml of tryptase (**a**) and chymase (**b**) and ATP levels were measured in cell culture supernatants after 1 h stimulation. Similarly, levels of uric acid were measured at 3 h (**c**) and 24 h (**d**). Data are presented as mean ± SEM, *n* = 4–12 from 3 to 6 independent experiments. *P < 0.05, **P < 0.01, ***P < 0.001, ****P < 0.0001 compared to respective control and ^#^*P* < 0.05, ^##^*P* < 0.01 compared to 0.1 μg/ml of tryptase

Extracellular ATP is considered an alarmin and is reported to be involved in allergen-driven airway inflammation [34, 35]. We have previously shown that allergen derived serine proteases induce release of ATP and uric acid in bronchial epithelial cells [35]. In this study, we investigated ATP and uric acid release in response to stimulation with MC serine proteases tryptase or chymase in HBECs. Here we show that dose dependent stimulation of both tryptase or chymase induced a rapid release of ATP in BEAS-2b (Fig. 1a and b) and primary HBECs (Supplementary Figure S1 A and B). However, we did not detect any uric acid release in response to tryptase or chymase stimulation compared to control (Fig. 1c and d).

### Mast cell proteases alter expression of airway epithelial barrier proteins

**Fig. 2:**
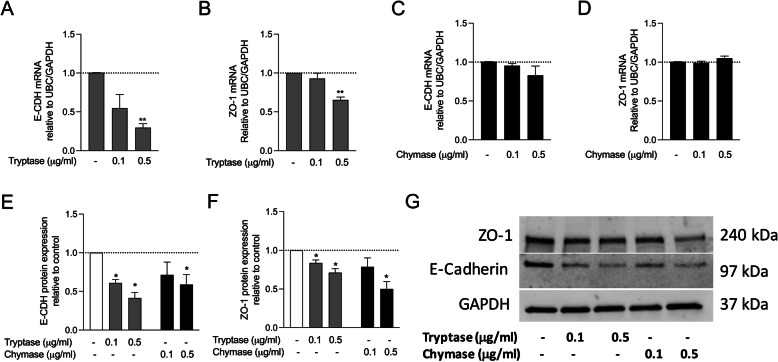
Mast cell proteases alter the expression of bronchial epithelial barrier proteins. HBECs were stimulated with tryptase or chymase and E-CDH gene expression (**a** and **c**) and protein expression (**e**) as well as ZO-1 gene expression (**b** and **d**) and protein expression (**e**) was investigated. Representative western blot protein expression (**f**). Data are presented as mean ± SEM, *n* = 4–6 from 6 independent experiments. *P < 0.05, **P < 0.01compared to respective control

Cell adhesion molecule E-CDH and tight junction protein ZO-1 are expressed by airway epithelial cells and are important for airway barrier functions [36]. RT-qPCR revealed significantly lower E-CDH (Fig. 2a) and ZO-1 (Fig. 2b) mRNA expression in HBECs after tryptase stimulation compared to control. However, no differences in E-CDH and ZO-1 mRNA expression were found comparing unstimulated and chymase stimulated HBECs (Fig. 2c and d). Western blot results showed that the protein expression of E-CDH (Fig. 2e, g) and ZO-1 (Fig. 2f, g) was downregulated when HBECs were stimulated with tryptase and chymase compared to controls.

### Tryptase induces release of pro-inflammatory cytokines and chemokines in bronchial epithelial cells

**Fig. 3:**
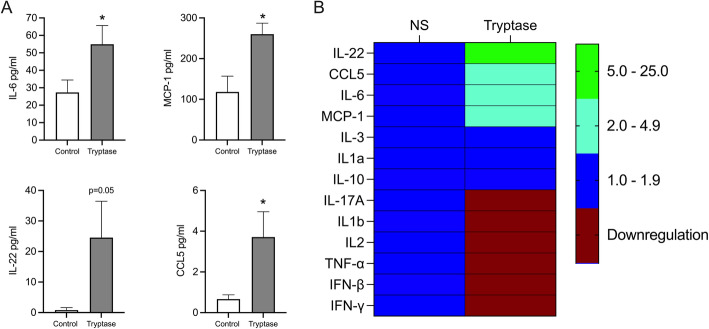
Tryptase stimulation induces release of pro-inflammatory mediators in HBECs. HBECs were treated with 0.5 μg/ml of tryptase and cell free supernatants were obtained after 24 h stimulation. Cytokines and chemokines levels were measured by multiplex Luminex. Significant increases of IL6, MCP-1, IL-22 and CCL5 were observed (**a**) and levels were expressed as mean ± SD in a heat-map (**b**). Levels of cytokines in the Luminex panel, CCL11, CD40 Ligand, Fractalkine, Flt-3, G-CSF, GM-CSF, Granzyme B, IL-1ra, IL-2, IL-4, IL-5, IL-7, IL-12, IL-13, IL-15, IL-17E, PD-L1 and TGF-α were not detectable. Data are presented as mean ± SEM, *n* = 4–8 from 3 independent experiments. *P < 0.05 compared to respective control

Next, we investigated if tryptase affected other mediators relevant to asthma by Luminex multiplex assay. Our data showed that tryptase alone significantly induced IL-6, IL-22, MCP-1 and CCL5 (Fig. 3a) but no further enhancement with poly(I:C) was observed for these mediators in HBECs. There was no effect of tryptase on CCL11, CD40 Ligand, Fractalkine, Flt-3, G-CSF, GM-CSF, Granzyme B, IL-1ra, IL-2, IL-4, IL-5, IL-7, IL-12, IL-13, IL-15, IL-17E, PD-L1 and TGF-α (Fig. 3b).

### Pre-treatment with tryptase or chymase enhances Poly (I:C) induced IL-8 in bronchial epithelial cells

**Fig. 4:**
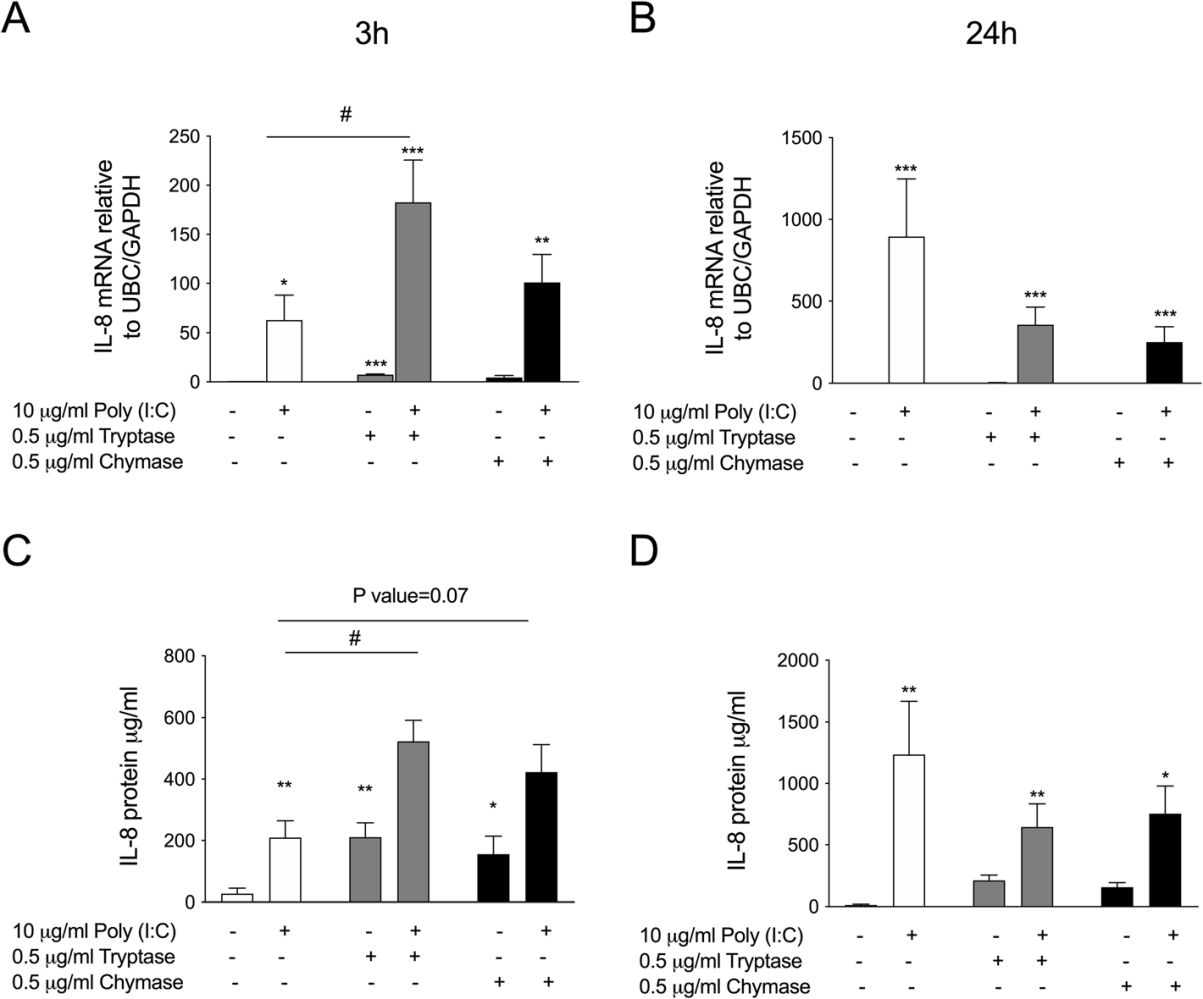
Pre-treatment with tryptase or chymase altered Poly (I:C) induced IL-8 release in HBECs. HBECs were pre-treated with tryptase or chymase for e hours and then stimulated with Poly (I:C) for 3 and 24 h. IL-8 mRNA expression 3 h (**a**) and 24 h (**b**). IL-8 protein release 3 h (**c**) and 24 (**d**). Data are presented as mean ± SEM, *n* = 4 from 4 independent experiments. *P < 0.05, **P < 0.01, **P < 0.001, ****P < 0.0001 compared to respective control and ^#^*P* < 0.05, ^##^*P* < 0.05 compared to Poly (I:C) alone

Viral infection induces augmented releases of pro-inflammatory mediators in asthmatic individuals [37]. Both tryptase and chymase dose-dependently induced IL-8 mRNA and protein release in BEAS-2b (Supplementary Figure S2 A-D). This observed increase in IL-8 protein release was confirmed in primary HBECs (Supplementary Figure S2 E). Additionally, we evaluated the effect of MC proteases on poly(I:C) induced pro-inflammatory mediator release. HBECs cells were pre-incubated with tryptase or chymase alone for 3 h, followed by stimulation with poly(I:C) for 3 and 24 h. IL-8 mRNA was induced by poly(I:C) and further enhanced in combination with tryptase (Fig. 4a). The same trend was observed for chymase. Furthermore, pre-treatment with tryptase or chymase enhanced poly(I:C) induced extracellular IL-8 protein release from HBECs (*p* = 0.07) (Fig. 4c). No significant effect of tryptase or chymase were observed at either mRNA or protein level at 24 h (Fig. 4b and d).

### Pre-treatment with tryptase decreases Poly (I:C) induced IFN-β and PRR expression in bronchial epithelial cells

**Fig. 5:**
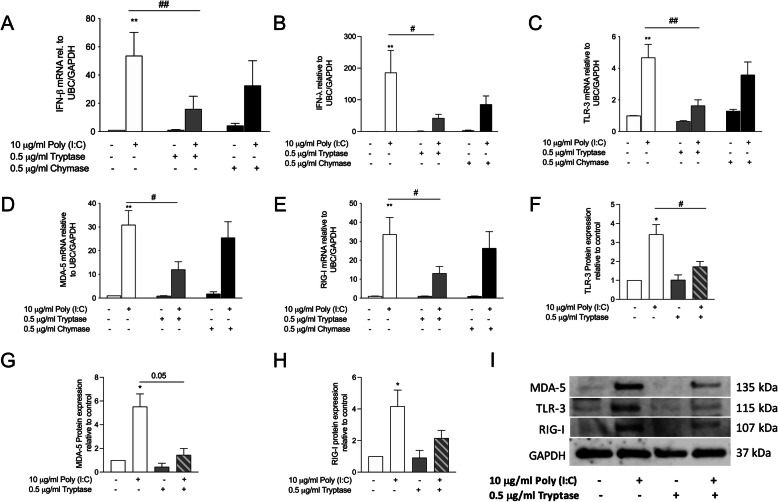
Pre-treatment with tryptase or chymase decreased Poly (I:C) induced IFN and PRR expression in HBECs. HBECs were pre-treated with tryptase or chymase followed by Poly (I:C) stimulation after 3 h. Gene expression was measured in cell lysate. Gene expression of IFN-β (**a**), IFN-lambda (**b**), TLR3 (**c**), MDA-5 (**d**) and RIG-I (**e**). Protein expression of TLR3 (**f**), MDA-5 (**g**) and RIG-I (**h**). Representative western blot of PRR protein expression at 24 h (**i**). Data are presented as mean ± SEM, *n* = 4 from 4 independent experiments. **P < 0.01 compared to respective control and ^#^*P* < 0.05, ^##^*P* < 0.05 compared to Poly (I:C) alone

The epithelium of asthmatic patients has been suggested to have an impaired anti-viral response [24, 38]. Recently we showed that house dust mite (HDM) impaired viral induced IFN response in HBECs [38]. Therefore, we examined if MC proteases, tryptase and chymase, can alter poly(I:C)-induced IFN response in bronchial epithelial cells. HBECs were pre-treated with tryptase or chymase for 3 h, then stimulated with poly(I:C) for 3 h and 24 h. Pre-treatment with tryptase significantly repressed the gene expression of type-I and III IFNs (Fig. 5a and b). Additionally, pre-treatment with tryptase significantly reduced expression of TLR3 and RIG-I like receptors MDA-5 and RIG-I at both gene (Fig. 5c-e) and protein levels (Fig. 5f-i). No significant effects on expression of IFN-β or PRRs was observed with pre-treatment with chymase. Our results showed that impaired effects of tryptase on poly(I:C)-induced epithelial IFN-β gene expression may be mediated by the interactions between mast cells proteases and PRR signalling pathways.

### Blocking of protease activity abolishes the tryptase induced impairment of IFN-β and PRR expression

**Fig. 6:**
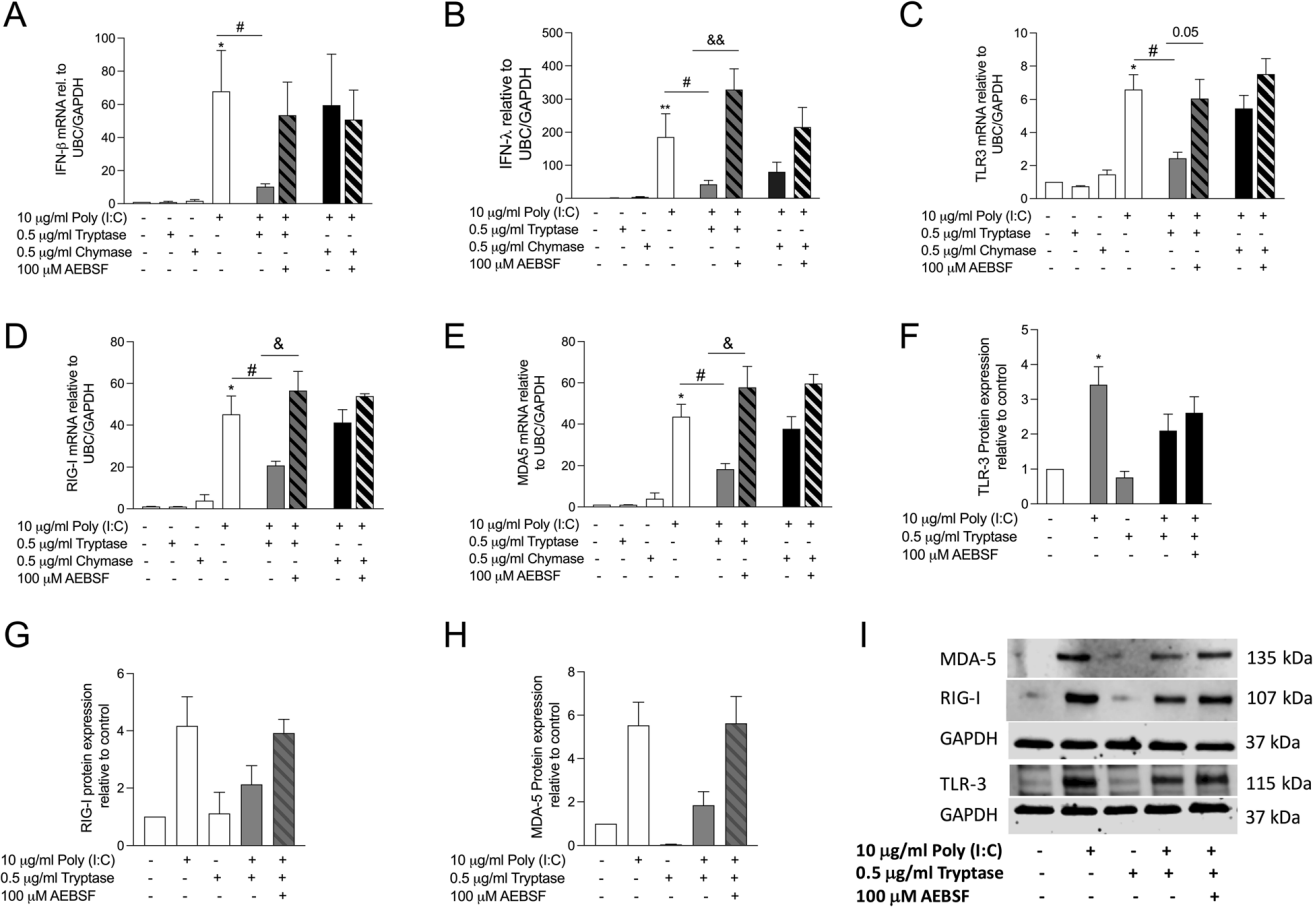
Blocking of protease activity abolish the Poly (I:C)-impairment of IFN and PRR expression by tryptase. Tryptase or chymase were pre-incubate with AEBSF, then pre-treated with tryptase or chymase alone or in combination with AEBSF followed by stimulation with Poly (I:C). Gene expression was measured in cell lysate. Gene expression of IFN-β (**a**), IFN-lambda (**b**), TLR3 (**c**), MDA-5 (**d**) and RIG-I (**e**). Protein expression of TLR3 (**f**), MDA-5 (**g**) and RIG-I (**h**). Representative western blot of PRR protein expression at 24 h (**i**). Data are presented as mean ± SEM, *n* = 4 from 4 independent experiments, *P < 0.05 compared to respective control, ^#^*P* < 0.05 compared to Poly (I:C) alone and ^&^*P* < 0.05 compared to combination with tryptase and Poly (I:C) and AEBSF

We have previously shown that the serine protease inhibitor AEBSF inhibited allergen-induced cytokine release [35]. Here we investigated whether the reduction of IFN-β and PRR expression by tryptase is due to the proteolytic mechanism of action of tryptase. Tryptase or chymase was pre-incubated together with the serine protease inhibitor AEBSF prior to stimulation. Our data showed that inhibiting tryptase with AEBSF restored the IFN-β and IFN- λ gene expression induced by poly(I:C) (Fig. 6a and b). Similar effects were found in PRR gene (Fig. 6c-e) and protein expression levels (Fig. 6f-i). These results suggest that the reduction of IFN-β and PRRs by tryptase is caused by its proteolytic action.

### Pre-treatment with tryptase decreases Poly (I:C) induced IFN-β and PRR gene expression in primary human bronchial epithelial cells

**Fig. 7:**
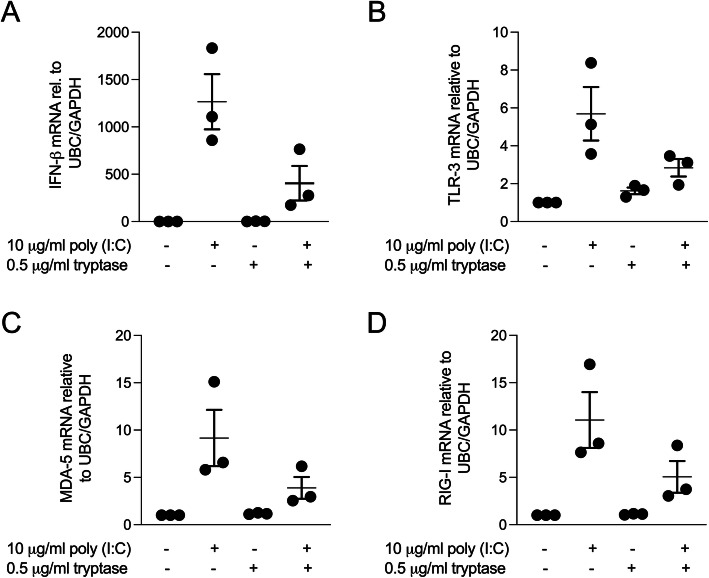
Pre-treatment with tryptase decreased Poly (I:C) induced IFNs and PRR gene expression in asthmatic primary HBECs. Primary HBECs were pre-treated with tryptase followed by stimulation with Poly (I:C). Gene expression was measured in cell lysate. Pre-treatment with tryptase impaired gene expression of poly (I:C) induced IFN-β (**a**), TLR-3 (**b**), MDA-5 (**c**) and RIG-I (**d**). Data are presented as mean ± SEM, *n* = 3 asthmatic donors

Next, in order to investigate if the observed effects of tryptase are valid in primary HBECs we performed the same experimental setup on primary HBECs from 3 asthmatic donors. Our preliminary data using human primary bronchial epithelial cells from asthmatic patients showed that pre-treatment with tryptase reduced poly(I:C) induced IFN-β, TLR3, MDA-5 and RIG-I gene expression (Fig. 7a-d).

## Discussion

In this study, we showed that stimulation of HBECs with MC proteases tryptase or chymase induce release of pro-inflammatory chemokine IL-8 and the alarmin ATP in vitro. Moreover, tryptase and chymase further elevated the pro-inflammatory response observed during stimulation with the viral mimic poly(I:C). Additionally, pre-treatment with tryptase impaired poly (I:C) induced type-I and III IFNs as well as PRR expression in HBECs in a protease dependent manner.

A majority, but not all studies of asthma have demonstrated an increase in numbers of MCs in the airway [2–7, 39], although very few studies account for the actual activity of MC mediator release. Elevated levels of MC serine proteases, tryptase and chymase are clinically important risk factors for chronic respiratory disease [21, 40]. To examine whether tryptase or chymase induce release of alarmins ATP and uric acid, HBECs were stimulated with tryptase or chymase. We found that both tryptase and chymase induced rapid release of ATP but not uric acid in HBECs and we propose that MC proteases can cause epithelial stress which leads to ATP release. We also investigated the effect of both tryptase and chymase on cytotoxicity levels in HBECs and did not observe any increase in cytopathic effects or LDH release in our cell cultures at 6 and 24 h suggesting that the alterations observed in this study were not caused by increased cell death (supplementary Fig. 3).

Tight junction proteins E-CDH and ZO-1 are major components of airway epithelial barrier. Although the usage of the immortalized BEAS-2b cell line in some studies has been shown to be sub-optimal for studying epithelial barrier function, other studies have shown that BEAS-2b cells express membrane bound ZO-1 and E-CDH [41]. Therefore, we evaluated the effect of MC proteases on epithelial barrier function in BEAS-2b. In our study, ZO-1 and E-CDH protein expression were significantly downregulated by both tryptase and chymase treatment. This data is in line with observations from Xiaoying Zhou et al., [42] who showed by immunostaining that disruption of cell junction proteins occur by chymase in 16HBE cells [41]. In concordance with the western blot analysis, mRNA expression of E-CDH and ZO-1 was downregulated by tryptase, but not by chymase. These findings indicate that increased number of MCs and their activation within the epithelium may cause epithelial barrier damage by degrading cell junctional molecules. Further studies are now needed to confirm these findings in primary epithelial cells grown in air liquid interface (ALI) system. Loss of epithelial barrier function can lead to increase viral load within the airway epithelium [43].

Previous studies have shown that tryptase induces production of pro-inflammatory mediators in airway structural cells and could therefore be important for the recruitment of inflammatory cells including neutrophils and eosinophils to sites of inflammation [44, 45]. It has been shown previously that tryptase can stimulate bronchial epithelial cell production of the pro-inflammatory chemokine IL-8 [7, 46] and we confirmed in the present study that both tryptase and chymase were involved in the induction of IL-8 gene and protein expression in HBECs. Furthermore, we investigated this pro-inflammatory response during a simultaneous stimulation with TLR3 agonist poly(I:C). We showed that MC proteases were responsible for the enhancement of poly (I:C) induced IL-8 production in HBECs. This result is in line with work by Zhu et.al., who showed that allergen proteases were major components to enhance the virus-induced cytokine productions in bronchial epithelial cells [47]. Synergistic interaction between allergic inflammation and viral infections are involved in asthma development as well as acute asthma exacerbations [48]. Our data indicates that interaction between mast cell proteases and epithelial cells could enhance the poly (I:C) induced inflammatory response in asthmatic airways, especially during a viral exacerbation. Patients with allergic airway diseases such as asthma, that are characterised by increased numbers of MCs in the mucosa, are therefore likely to have an augmented inflammatory response when infected by viruses.

In the present study, we found that pre-treatment with tryptase impaired type-I and III IFN expression by the viral mimic poly(I:C) in both BEAS-2b and asthmatic primary HBECs. This finding is in line with our previous finding that pre-exposure to HDM allergen impairs the type-I and III IFN response to poly(I:C) both in vitro and in vivo [38]. In a similar way, MC proteases impaired anti-viral responses in HBECs, which may have an important role in the immune defence to respiratory viruses. Furthermore, we found that a serine protease inhibitor restored the IFN expression in bronchial epithelial cells, strongly suggesting that the effect of tryptase was mediated through a proteolytic mechanism. Moreover, we demonstrated a mechanism by which tryptase reduced the Poly(I:C) induced IFN production: by downregulating expression of PRRs TLR3, RIG-I and MDA-5. This data supports the hypothesis that MC proteases altered poly(I:C)-induced immunity in an imbalanced manner, in which IFN and PRR expression was decreased. The presence of activated MCs within the airway mucosa may therefore impair important epithelial viral defence mechanisms.

Although Poly(I:C) does not evoke completely the same response in bronchial epithelial cells as real rhinovirus, several studies have demonstrated that intracellular transduction of poly(I:C) mediates a strong proinflammatory and anti-viral response in airway epithelial cells with respect to cytokine, chemokine and IFN secretion. The strong inflammatory properties of the TLR3 ligand poly(I:C) have thus shown to activate bronchial epithelial cells in a way that mimics rhinovirus-induced exacerbations observed in pulmonary diseases like asthma [38, 49, 50]. Furthermore, by using poly(I:C) one can avoid variations in rates of infection, thus, enhancing the reproducibility of the data. This is particularly important in our study since it is the first time the anti-viral response is studied in combination with mast cell proteases. However, future studies should focus on the use of live virus to also investigate the cytopathic effect of a replicating virus.

Although both tryptase and chymase are serine proteases, we observed different responses in HBECs, particularly in the anti-viral response where chymase had less effect. This different mechanism of action could suggest specialized functions of the different protease structures and their interaction with different cell types. A proposed model which would be further supported by the heterogeneity and different localisation of MC subtypes, as tryptase positive mast cells (MC_T_) and not tryptase and chymase positive mast cells (MC_TC_), _are_ found within the epithelium in numerous different airway pathologies. Further studies are warranted to elucidate how the different mechanism of actions of tryptase and chymase could be connected to their localisation within the airways and how these proteases may alter the PRR expression in HBECs.

## Conclusions

In conclusion, we showed that MC proteases tryptase and chymase induced secretion of the metabolic alarmin ATP and pro-inflammatory mediators IL-8 and IL-6 in HBECs. Additionally, we demonstrated, for the first time, that anti-viral responses (TLR signalling and IFN production) in HBECs are impaired by MC tryptase, and that this is dependent on the proteolytic activity of tryptase. Our data may partly give a mechanistic explanation as to why individuals with atopic asthma and increased numbers of mucosal MCs are more prone to develop virally induced exacerbations.

## Methods

The aim of this study is to investigate the direct effects of stimulation with MC proteases, tryptase and chymase, on inflammatory and anti-viral responses in HBECs.

### Bronchial epithelial cell cultures, stimulations and treatments

The human bronchial epithelial cell line, BEAS-2b was purchased from ATCC (Walkersville, MD, USA) and maintained in RPMI 1640 medium supplemented with 10% foetal bovine serum (FBS) and 1% Penicillin and streptomycin from Life Technologies (Stockholm, Sweden) as previously described [51]. Primary HBECs were collected from three steroid treated mild asthmatic patients (mean age 35, range [30–38]) that underwent clinical bronchoscopy with endobronchial brushings and expanded and cultured according to previous validated protocols [52]. Two out of three patients were atopic and had treatment with short acting β2 agonists, all subjects had normal FEV_1_% predicted (mean 89, range [83–98]). Cells were seeded in collagen coated 12-well culture plates and grown to 80–90% confluency [35] before being stimulated with different concentrations of human lung tryptase; 0.1 and 0.5 μg/ml protein with specific protease activity 62.5 U/mg (Merck Millipore, Darmstadt, Germany) and chymase; 0.1 and 0.5 μg/ml of protein with specific protease activity 60–90 U/mg (Sigma-Aldrich, Stockholm, Sweden) for 1, 3, 6 and 24 h. To investigate effects of tryptase and chymase on the anti-viral immune response, bronchial epithelial cells where first pre-treated with tryptase and chymase for 3 h followed by TLR3 stimulation for 3 and 24 h using Poly(I:C), to mimic a rhinovirus infection [52]. In separate experiments, optimal concentrations of tryptase and chymase stimulation were pre-incubated with 100 μM of a serine protease inhibitor 4-(2-aminoethyl) benzene sulfonyl fluoride hydrochloride (AEBSF) (Sigma-Aldrich, Stockholm, Sweden) as previously reported [38]. Cell lysate and cell-free supernatant were collected for further analysis.

### ATP measurements

ATP Kit SL (BioThema luminescent assay, Handen, Sweden) was used to analyse ATP levels in cell supernatants according to manufacturer’s instruction and as previously described [35].

### LDH measurements

Levels of lactate dehydrogenase were measured in cell-free supernatants according to the manufacturer’s instructions (Roche Diagnostics, Bromma, Sweden) and related to total protein content in the supernatant (Pierce, Thermo Scientific, Waltham, MA, USA) and as previously described [35].

### RNA isolation and real-time qPCR

Total RNA was extracted from BEAS-2b cells using an RNA extraction kit, Nucleospin RNA II (Machery-Nagel, Düren, Germany), according to the manufacturer’s instructions and as previously described [35]. 1 μg of RNA was reverse transcribed to cDNA (Precision Nanoscript Reverse Transcription Kit, PrimerDesign, Southampton, UK) and real-time quantitative PCR was performed using an Mx3005P qPCR system (Stratagene, La Jolla, CA, USA) with standard cycling parameters using the primer and probes of interest. The following primer sequences (PrimerDesign, Southampton, UK) were used: human RIG-I: TTCTCTTGATGCGTCAGTGATA (forward) and CCGTGATTCCACTTTCCTGAA (reverse), human MDA5: GTCTCGTCACCAATGAAATAGC (forward) and TTATACATCATCTTCTCTCGGAAATC (reverse), human TLR3: GTGTGAAAGTATTGCCTGGTTTGT (forward) ATGATAGTGAGGTGGAGTGTTGC (reverse), human IFNβ: TTACTTCATTAACAGACTTACAGGT (forward) and TACATAGCCATCGTCACTTAAAC (reverse), IFN-λ_1_: ATGGGAACCTGTGTCTGAGAA (forward), and GGGTGAGAGGAAATAAATTAAGGAA (reverse) and CXCL8: CAGAGACAGCAGAGCACAC (forward) and AGCTTGGAAGTCATGTTTACAC (reverse). Quantitative normalization was performed on the expression of ubiquitin C (UBC) and glyceraldehyde 3-phosphate dehydrogenase (GAPDH). Group comparisons were normalised to control sample using the comparative ^△△^Ct method [53].

### Western blot analysis

Protein expression of E-CDH, ZO-1, TLR3, MDA5 and RIG-I was quantified by Western blot. Experiments were performed as described in previous sections and cells were lysed 24 h after treatment using a lysis buffer containing 1% TritonX-100, 10 mM Tris-HCl, 50 mM NaCl, 5 mM EDTA, 30 mM Na4P2O7, 50 mM NaF, 0.1 mM Na3VO4, 1% phosphatase and protease inhibitors (Sigma-Aldrich, Stockholm, Sweden). Protein concentrations were determined by BCA protein assay (Pierce Thermo Scientific, Waltham, MA, USA) for each sample and equal amounts of protein were loaded and electrophoresed on a 10% TGX stain-free gel (Bio-Rad Laboratories AB, Solna, Sweden), before blotting on a Trans-Blot Turbo Transfer System (Bio-Rad Laboratories AB, Solna, Sweden). This was followed by blocking of the membrane in 5% (w/v) milk in Tris-buffered saline Tween-20 and overnight incubation at 4 °C with primary antibodies TLR3, MDA5 and RIG-I (Cell Signalling Technology, Leiden, The Netherlands); E-CDH (Dako, and ZO-1 (Invitrogen, Gothenburg, Sweden). The membrane was washed and incubated for 1 h with secondary antibodies (anti-mouse IgG HRP-linked Ab; Cell signalling Technology, Leiden, The Netherlands). Detection was performed by chemiluminescence using SuperSignal West FEMTO substrate (Pierce Thermo Scientific, Waltham, MA, USA) and immunoblots were visualized by LI-COR Odyssey Fc Imager (LI-COR Biosciences, Lincoln, NE, USA) and Image Studio (v.3.1.4; LI-COR Sciences, Lincoln, NE, USA).

### ELISA analysis of IL-8 protein release in cell supernatants

IL-8 levels were measured in cell supernatants using a specific ELISA with matched antibodies from R&D Systems (Minneapolis, USA) according to the manufacturer’s descriptions.

### Protein quantification by multiplex Luminex

Levels of cytokines MCP-1, CCL5, CCL11, CD40 Ligand, Fractalkine, Flt-3, G-CSF, GM-CSF, Granzyme B, IFN-α, IFN-β, IFN-γ, IL-1 α, IL-1 β, IL-1ra, IL-2, IL-3, IL-4, IL-5, IL-6, IL-7, IL-8, IL-10, IL-12, IL-13, IL-15, IL-17A, IL-17E, PD-L1, TGF-α and TNF-α were measured in cell-free supernatants by Luminex immunoassay (customized Human XL Cytokine Luminex Performance Panel, FCSTM18, R&D Systems, Abingdon, UK) according to the manufacturer’s instructions. Data was acquired on a calibrated and validated Luminex MAGPIX instrument (R&D Systems, Abingdon, UK) as per manufacturer’s instructions.

### Statistical analysis

Data were analysed using the software GraphPad Prism version 6.0 (San Diego, CA, USA) and expressed as mean value with SEM. Significant variations between the groups were analysed using ANOVA followed by Dunn’s multiple comparison test, while the Mann–Whitney test was used to analyse differences between unpaired groups. *P* values of less than 0.05 were considered statistically significant.

## Supplementary Information

**Additional file 1: Supplementary Figure S1.** Mast cell proteases induce alarmin ATP release in primary HBECs. Primary HBECs were treated with 0.5 μg/ml of protein concentrations of tryptase or chymase. ATP levels were measured in cell culture supernatants. Tryptase (A), and chymase (B) for 1 h. Data are presented as mean ± SEM, *n* = 3 asthmatic donors. **Supplementary Figure S2.** IL-8 gene expression and protein release was increased by tryptase and chymase. HBECs were treated with 0.1 μg/ml of protein and 0.5 μg/ml of protein, tryptase or chymase for 6 h and 24 h. IL-8 gene expression Tryptase (A) and Chymase (C). IL-8 protein levels in supernatants Tryptase (B) and Chymase (D). IL-8 protein levels in primary HBECs from asthmatic patients (E), *n* = 3. Data are presented as mean ± SEM, *n* = 6–7 from 7 independent experiments. *P < 0.05, **P < 0.01 compared to respective control and ^#^P < 0.05 compared to 0.1 μg/ml of protein. ND- Non-detected. **Supplementary Figure S3.** LDH release by tryptase and chymase. HBECs were treated with 0.1 μg/ml of protein and 0.5 μg/ml of protein, tryptase or chymase. LDH release in cells supernatant at 6 h (A) and 24 h (B). Data are presented as mean ± SEM.

## Data Availability

The datasets during and/or analysed during the current study available from the corresponding author on reasonable request.

## Abbreviations

MCs: Mast cells
HBECs: Human bronchial epithelial cells
PRRs: Pattern recognition receptors
TLRs: Toll like receptors
IFN-β: Interferon beta
IFN-λ: Interferon lambda
Poly (I:C): Polyinosinic:polycytidylic acid
ATP: Adenosine triphosphate
LDH: Lactate dehydrogenase
E-CDH: E-cadherin
ZO-1: Zonula occludens-1
MC_T_: Tryptase positive mast cells
MC_TC_: Tryptase and chymase positive mast cells
AEBSF: 4-(2-aminoethyl) benzenesulfonyl fluoride hydrochloride
