# Supplementary material for: Direct effects of mast cell proteases, tryptase and chymase, on bronchial epithelial integrity proteins and anti-viral responses
Source: BMC Immunol. 2021 Jun 2;22:35. doi: 10.1186/s12865-021-00424-w (PMC8170739; doi:10.1186/s12865-021-00424-w)
Supplement: Supplementary file 1 — Additional file 1: Supplementary Figure S1. Mast cell proteases induce alarmin ATP release in primary HBECs. Primary HBECs were treated with 0.5 μg/ml of protein concentrations of tryptase or chymase. ATP levels were measured in cell culture supernatants. Tryptase (A), and chymase (B) for 1 h. Data are presented as mean ± SEM, n = 3 asthmatic donors. Supplementary Figure S2. IL-8 gene expression and protein release was increased by tryptase and chymase. HBECs were treated with 0.1 μg/ml of protein and 0.5 μg/ml of protein, tryptase or chymase for 6 h and 24 h. IL-8 gene expression Tryptase (A) and Chymase (C). IL-8 protein levels in supernatants Tryptase (B) and Chymase (D). IL-8 protein levels in primary HBECs from asthmatic patients (E), n = 3. Data are presented as mean ± SEM, n = 6–7 from 7 independent experiments. *P < 0.05, **P < 0.01 compared to respective control and #P < 0.05 compared to 0.1 μg/ml of protein. ND- Non-detected. Supplementary Figure S3. LDH release by tryptase and chymase. HBECs were treated with 0.1 μg/ml of protein and 0.5 μg/ml of protein, tryptase or chymase. LDH release in cells supernatant at 6 h (A) and 24 h (B). Data are presented as mean ± SEM. [file 12865_2021_424_MOESM1_ESM.docx]

**Supplemental materials**

**Direct effects of mast cell proteases, tryptase and chymase, on bronchial epithelial integrity proteins as well as cytokine and anti-viral responses**

**Sangeetha Ramu^1^, Hamid Akbarshahi^1,2^, Sofia Mogren^1^, Frida Berlin^1^, Samuel Cerps^1^, Mandy Menzel^1^, Morten Hvidtfeldt^3^, Celeste Porsbjerg^3^, Lena Uller*^1^ and Cecilia K Andersson*^1^**

*^1^Dept of Experimental Medical Science, Lund University, Lund, Sweden*

*^2^Dept of Respiratory Medicine and Allergology, Lund University, Lund, Sweden*

*^3^Department of Respiratory Medicine, Bispebjerg and Frederiksberg Hospital, Copenhagen, Denmark*

**These authors contributed equally*

**Correspondence to:** Cecilia Andersson

Department of Experimental Medical Science

Lund University

Lund, Sweden

Tel: +46 222 77 46

E-mail: [cecilia.andersson@med.lu.se](mailto:cecilia.andersson@med.lu.se)

**Running head:** Effects of mast cell proteases on bronchial epithelial cells


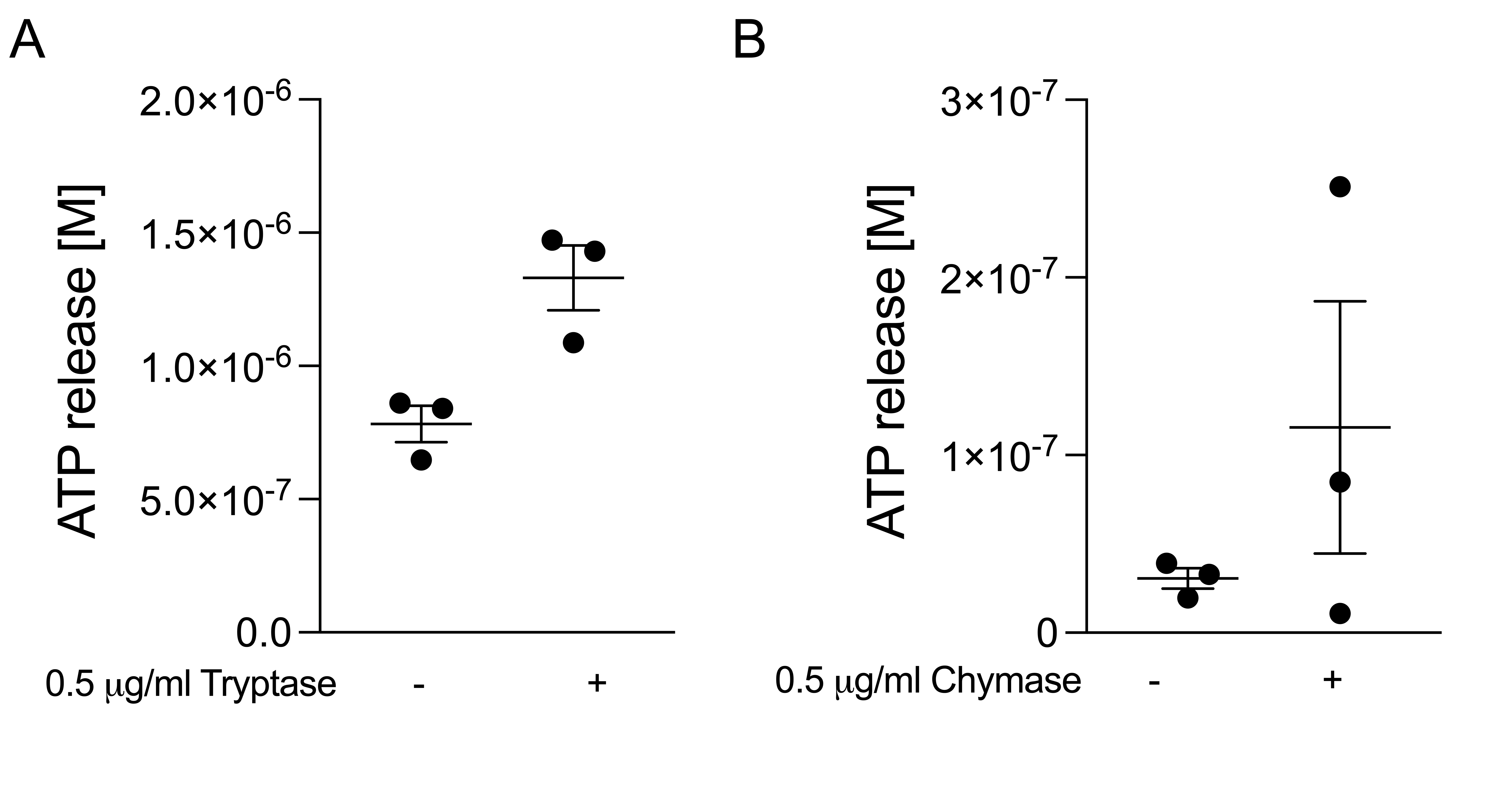


**Supplementary Figure S1: Mast cell proteases induce alarmin ATP release in primary HBECs.** Primary HBECs were treated with 0.5 μg/ml of protein concentrations of tryptase or chymase. ATP levels were measured in cell culture supernatants. Tryptase (**A**), and chymase (**B**) for 1h. Data are presented as mean ± SEM, n=3 asthmatic donors.


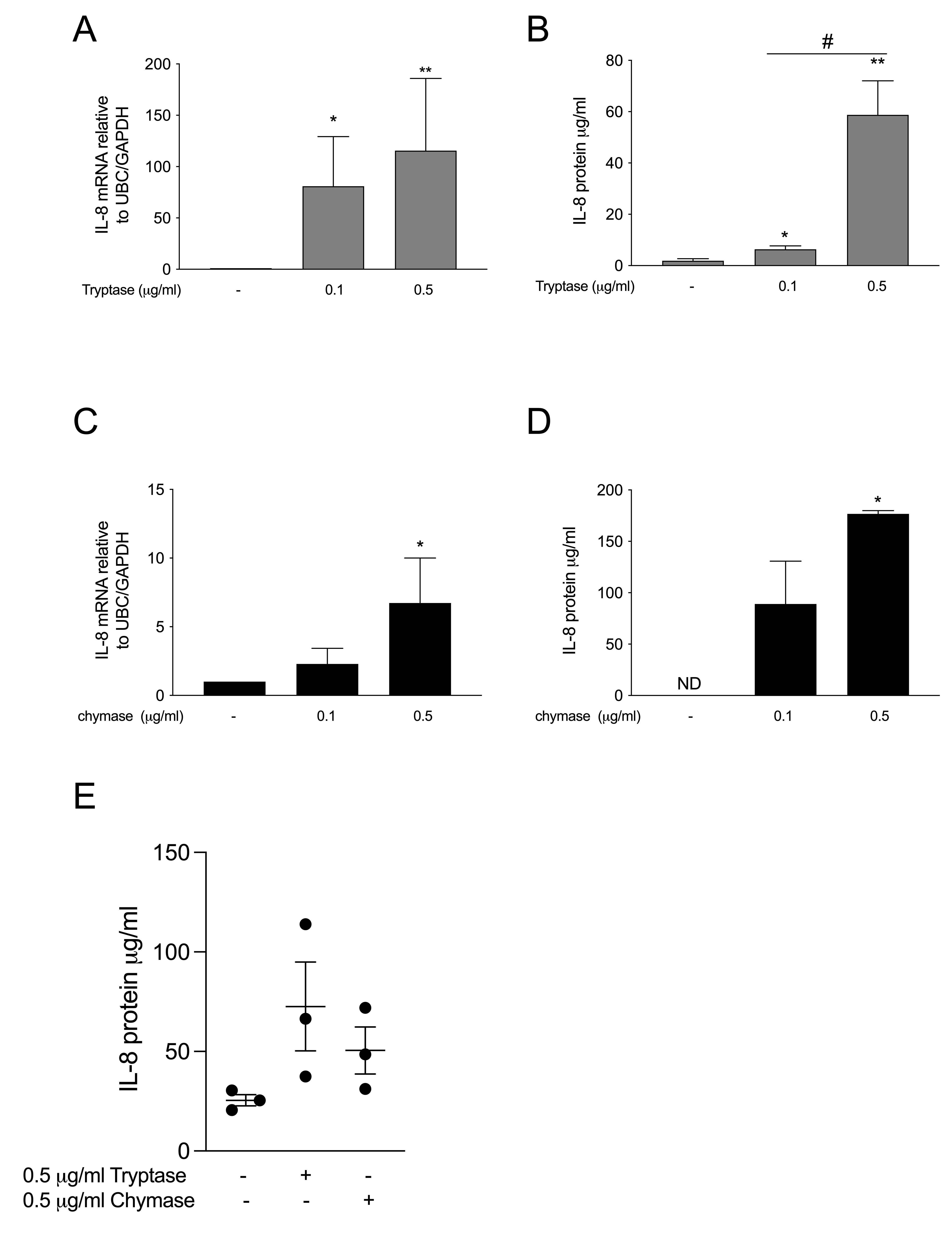


**Supplementary Figure S2.** **IL-8 gene expression and protein release was increased by tryptase and chymase**. HBECs were treated with 0.1 μg/ml of protein and 0.5 μg/ml of protein, tryptase or chymase for 6h and 24h. IL-8 gene expression Tryptase (**A**) and Chymase (**C**). IL-8 protein levels in supernatants Tryptase (**B**) and Chymase (**D**). IL-8 protein levels in primary HBECs from asthmatic patients (**E**), n=3. Data are presented as mean ± SEM, n= 6-7 from 7 independent experiments. *P< 0.05, **P<0.01 compared to respective control and ^#^P<0.05 compared to 0.1 μg/ml of protein. ND- Non-detected.


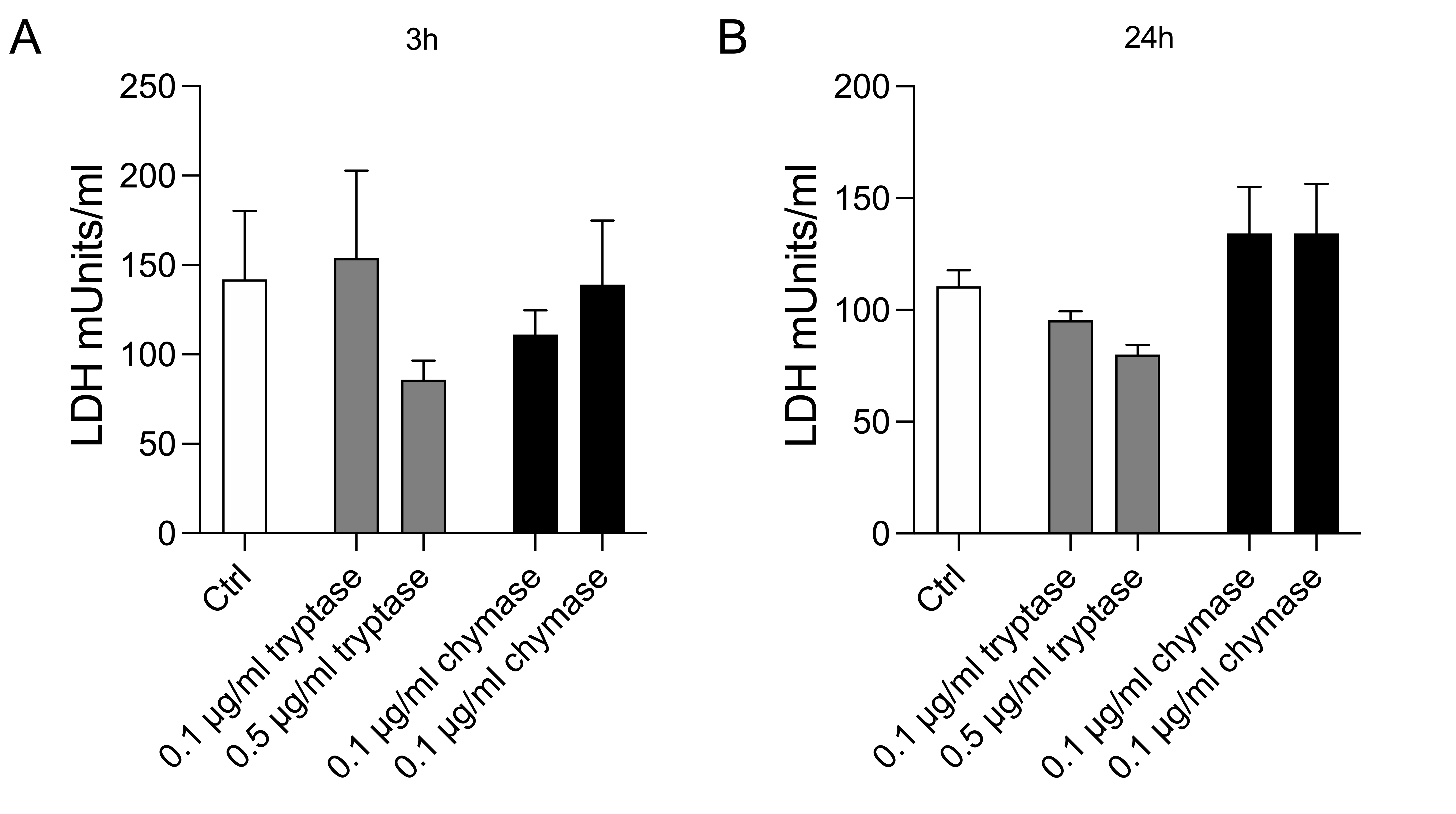


**Supplementary Figure S3.** **LDH release by tryptase and chymase**. HBECs were treated with 0.1 μg/ml of protein and 0.5 μg/ml of protein, tryptase or chymase. LDH release in cells supernatant at 6h (**A**) and 24h (**B**). Data are presented as mean ± SEM.
